# A single respiratory tract infection early in life reroutes healthy microbiome development and affects adult metabolism in a preclinical animal model

**DOI:** 10.1038/s41522-022-00315-x

**Published:** 2022-07-02

**Authors:** Soner Yildiz, Beryl Mazel-Sanchez, Joao P. P. Bonifacio, Mirco Schmolke

**Affiliations:** 1 Department of Microbiology and Molecular Medicine, Faculty of Medicine, University of Geneva, Geneva, Switzerland; 2 Geneva Center for Inflammation Research, Geneva, Switzerland

**Keywords:** Microbiome, Pathogens

## Abstract

In adult animals, acute viral infections only temporarily alter the composition of both respiratory and intestinal commensal microbiota, potentially due to the intrinsic stability of this microbial ecosystem. In stark contrast, commensal bacterial communities are rather vulnerable to perturbation in infancy. Animal models proved that disruption of a balanced microbiota development e.g., by antibiotics treatment early in life, increases the probability for metabolic disorders in adults. Importantly, infancy is also a phase in life with high incidence of acute infections. We postulated that acute viral infections in early life might pose a similarly severe perturbation and permanently shape microbiota composition with long-term physiological consequences for the adult host. As a proof of concept, we infected infant mice with a sub-lethal dose of influenza A virus. We determined microbiota composition up to early adulthood (63 days) from small intestine by 16S rRNA gene-specific next-generation sequencing. Infected mice underwent long-lasting changes in microbiota composition, associated with increase in fat mass. High-fat-high-glucose diet promoted this effect while co-housing with mock-treated animals overwrote the weight gain. Our data suggest that in the critical phase of infancy even a single silent viral infection could cast a long shadow and cause long-term microbiota perturbations, affecting adult host physiology.

The establishment of a stable adult-like intestinal microbiota takes three to four years in humans (reviewed in^1^). In laboratory mice, this phase takes about three weeks^2^, due to the higher developmental speed, and is largely driven by the change in nutrition from milk to solid chow. Interference with microbial ecology in this early phase, e.g., by administrating antibiotics (ABX), results in a long-term dysbiotic state and increased incidence of obesity and type II diabetes both in humans^3,4^ and mice^5^. Of note, the time frame between ABX treatment and clinical readout was limited in human observational studies to a maximum of 12 years.

Infancy is also a phase in human life with increased incidence of acute infections. From previous experiments we knew, that an established and robust adult intestinal microbiota, returns to baseline after undergoing qualitative and quantitative changes in consequence of an acute infection, e.g., with influenza A virus^6–9^. We hypothesized that a single acute infection event, might be sufficient to permanently perturb commensal bacterial composition, when occurring in a dynamic and vulnerable phase of microbiota development. As a proof of concept, we demonstrate here that infant mice infected with influenza A virus (IAV) undergo a microbiota imprinting event, with an elevated risk for long-lasting qualitative and quantitative changes in intestinal microbiota composition and overall animal growth.

Infection of 7d- old (D7), infant C57BL/6J mice with a single sublethal dose (40pfu) of influenza A virus A/Viet Nam/1203/2004 Halo^10^, which resulted in robust viral replication restricted to the upper and lower respiratory tract (as determined by plaque assay (Fig. 1a and Fig. S1a), had no measurable impact on early life body weight development (Fig. 1b). This dose was previously used to sub-lethally infect adult animals^6^ and surprisingly did not cause differences in weight gain here infant mice. Our finding contrasts observations made with mouse-adapted H1N1 virus, which is more lethal in neonate mice^11^. We thus consider our model as a subclinical infection model, mimicking a mild childhood disease in humans. Viral titers were cleared by 14d post-infection, comparable to viral kinetics in adult animals^6^. We next used 16S rRNA gene-specific next-generation sequencing (NGS) as described previously^6^ on total DNA from small intestinal tissue homogenates from day 7, 14, and 56-post viral infection. Mice were infected in at least two independent experiments with at least two independent cages per run. In adult animals, we previously described quantitative reduction of small intestinal microbiota in IAV-infected animals, associated with a reduced alpha diversity and a shift in beta diversity. At the same time, we did not find changes in fecal microbiota diversity^6^. Similar to these results from adult mice infected with IAV, infant mice displayed significantly reduced alpha diversity 7d post-infection, but not at later time points (Fig. 1c). Additionally, we found overall ~5-fold reduced 16 S/18 S levels^6^ in the small intestine of D56 mice infected with IAV in infancy, indicating a long-term reduction in bacterial colonization by viral imprinting (Fig. 1d). Of note by D14 post-infection we already determined a reduced 16S/18S ratio, suggesting that this development starts during or right after clearance of the virus. When comparing microbiota composition on day 7-post infection, we found predominance of Bacilli both in mock-treated and IAV-infected animals, which is likely a consequence of breast milk-based nutrition (Fig. S2a). The drop in alpha diversity and the relative overrepresentation of Lactobacilli could be explained by a loss of low abundance populations, present in mock-treated mice (Fig. S2a/b). 14 days post-treatment (age 21d) mice showed diversified intestinal microbiota, with all major classes present in adult microbiota (Fig. S2a), most likely to the introduction of solid food into the diet and the lack of continuous milk intake. LefSe analysis^12^ revealed significant increase in Gammaproteobacteria in IAV-infected mice at this stage (Fig. S2c). By day 56-post infection (9-weeks-old mice) we found Enterobacteriaceae significantly increased in IAV-infected mice. Conversely, in mock-treated animals several groups of Firmicutes (mainly of the class Clostridia) were enriched. (Fig. 1e) Intriguingly, in mice, Enterbacteriaceae are favored under high-fat diet conditions^13^ or after low dose penicillin treatment, which both lead to obesity in mice^5^. In line with our findings from adult mice, in two American studies, populations of obese patients had enriched Enterobacteriaceae and decreased Clostridia^14^. In accordance with the compositional data, weighed PCoA analysis revealed a more dispersed beta diversity for mock-treated animals on D7 post-infection (Fig. 1f). By D14 post-infection, no obvious differences between the two mouse groups were apparent. Interestingly, a higher divergence along PC1 and PC2 was found for IAV-infected animals on D56 post-infection, which does not reach statistical significance, probably as a consequence of the large variance within this group (see Permadisp analysis in Supplementary Table 1). A direct comparison of D56 mock and IAV treated mice by Permanova analysis indicated a *p*-value of 0.178. Since we cannot monitor the severity of the infection in the young mice, due to lack of clinical signs, we propose that variability of infection could explain why few of the infected animals cluster with the majority of the mock-treated mice (bottom right corner of the graph in Fig. 1f). While IAV does not replicate in the intestine an increased interferon response was proposed to trigger microbiota changes in the intestinal tract after IAV infection^8^. We confirmed the absence of viral RNA in the small intestine by highly sensitive qPCR (Fig. S2d). Intriguingly we found 2fold-increased levels of IL28 mRNA but no changes in IP10 mRNA in the intestine of mice infected with IAV (Fig. S2e). This could explain the alterations of microbiota diversity and composition. At this stage, we speculate that the loss of rare OTUs might create a bottleneck early in life, which results in different trajectory of microbial community development. Likely, both direct and indirect mechanism play a role in this alteration. Obviously, this would open a therapeutic window for correction of infection-dependent microbiota depletion, e.g., by targeted application of probiotics. Strikingly, as previously shown in adult animals^6^, fecal microbiota were completely resistant to infection-related imprinting effects as shown by indistinguishable alpha and beta diversity and only minor differences in composition (Fig. S3a–c).

**Fig. 1:**
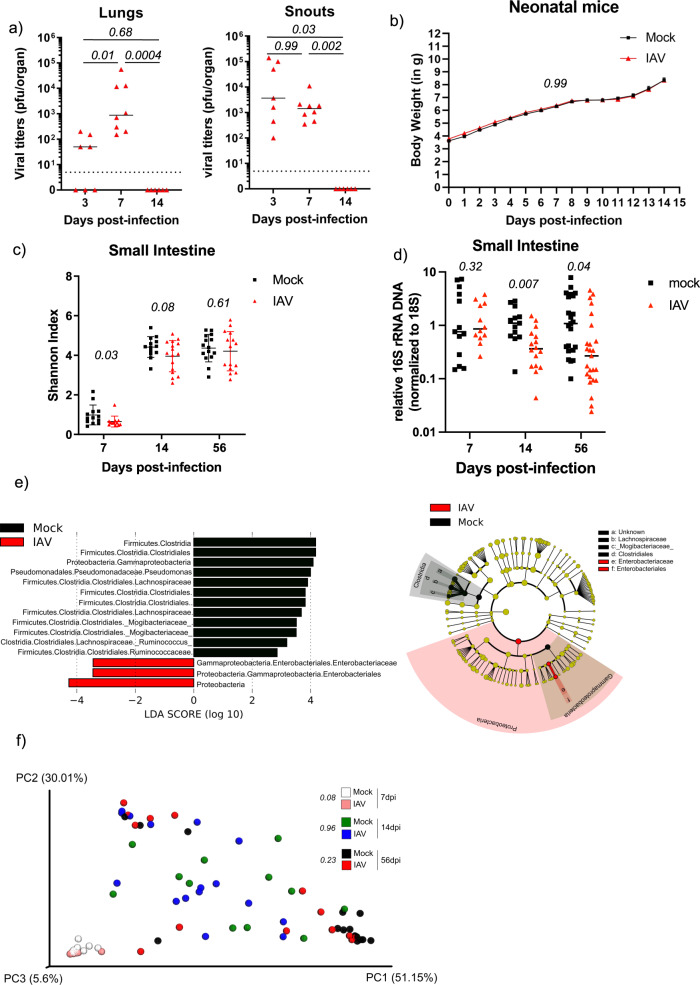
Neonatal IAV infection changes microbiota composition in adult mice. Seven-days-old mice were infected intranasally with PBS or 40 pfu of VN/1203 in 5 µL of PBS. **a** At 3-, 7- and 14 dpi viral titers in lungs and snouts for IAV infected mice were determined by plaque assay. Each dot represents one animal (*n* = 7–8) and the median titer is represented by a black dash. Limit of detection is represented by a black dashed line. Statistical significance between groups was determined by Kruskal–Wallis test. *P*-values are indicated. **b** Body weight was followed for 14 days after infection and values for male mice are plotted as mean ± SEM (*n* = 29 for mock-treated and *n* = 38 for IAV-infected). Statistical significance between groups was determined by 2-way Anova test. *P*-value is indicated. **c** Individual Shannon H-index of small intestine microbiota at 7, 14, and 56dpi of mock-treated (black squares) and IAV-infected mice (red triangles) are depicted for indicated days of sampling. Mean ± SD per experimental group are shown. Statistical significance between groups was determined by unpaired students’ *t*-test. *P*-values are indicated. **d** Normalized individual 16 S/18 S qPCR results (n-fold relative to mean of mock samples) in small intestine at 7, 14, and 56 dpi are depicted for mock-infected (black squares) and IAV-infected (red triangles). Mean ± SD per experimental group are shown. Statistical significance between groups was determined by unpaired students’ *t*-test. **e** LefSe analysis of the composition of the microbiota in small intestines of mock-treated and IAV-infected mice based on 16S rRNA gene sequencing of samples collected at 56dpi. Linear discriminant analysis scores (LDA) are indicated for different taxonomic groups significantly overrepresented (*p* < *0.05*) in mock-treated or IAV infected mice. (*n* = 15). Kruskall–Wallis statistical test was performed as described previously^12^. Cladograms of OTUs, as annotated by Qiime1, that are differentially represented in small intestine samples taken from mock and IAV infected animals on the right-hand side. Overrepresented taxonomy groups are given on legends next to the corresponding cladogram. **f** Scaled 3D principal coordinates analysis (PCoA) plots using a weighted-UniFrac distance matrix from small intestine microbiota of mock-treated or IAV-infected mice at indicated time points post-infection. Each symbol represents one individual mouse. Percentages explain variation in PC1 (x-axis), PC2 (y-axis) and PC3 (z-axis). PERMANOVA and PERMDISP statistical tests were performed and p-values are indicated in the figure and listed in Supplementary Table 1.

Intestinal microbiota are a driving force of host physiology (reviewed in^15^). In adult mice, acute IAV infection causes metabolic changes in adipose tissues, which eventually last until after IAV clearance^16^. We thus asked whether the long-term changes in small intestinal microbiota composition after early-life infection indeed affect the metabolism of adult mice. A striking observation arose from adult body weight curves. While there was overall no difference in the weight development of infected vs. mock-treated mice, gender-stratified data revealed a clear increase of absolute body weight in adult male mice infected with IAV in early in life starting around D42 post-infection (Fig. 2a), adding up to a median of about 3% body weight gain by D56 post-infection. We observed overall a similar tendency but with more variation in body weight among female mice, potentially due to the unsynchronized hormonal cycle of these animals (Fig. S4a). It further has to be taken into account, that female mice respond generally weaker to e.g., diet-induced weight gain^17^. Hence, we focused on male mice to further address the observed weight gain phenotype. Since both decreased activity or increased food uptake or processing could be a reason for the increase weight, we performed metabolic phenotyping of individually caged male mice 56d post-infection using TSE Labmaster system (TSE Systems, Germany). We found no difference in movement patterns, food, and water intake, or fatty acid oxidation^18^, which could have explained increased body weight (Fig. S4b–f). Similarly, caloric uptake, as determined by bomb calorimetry of consumed food and shed fecal matter, yielded no differences between the two mouse groups **(**Fig. S4g**)**. We did, however, observe a decrease in specific energy expenditure (determined by indirect calorimetry^19^) after IAV infection, both during day- and night-time which could explain in part the increase body weight (Fig. 2b). We next determined lean and fat mass by Echo-MRI, and found a tendency for increased total fat mass in previously IAV-infected mice (*p* = *0.13*) (Fig. 2c). Acute IAV infection was recently shown to affect white fat browning^16^. However, analysis of fat browning-related marker genes did not reveal important differences in mRNA expression as determined by specific qPCR (Fig. S4h). This makes involvement of reduced-fat browning in weight gain unlikely.

**Fig. 2:**
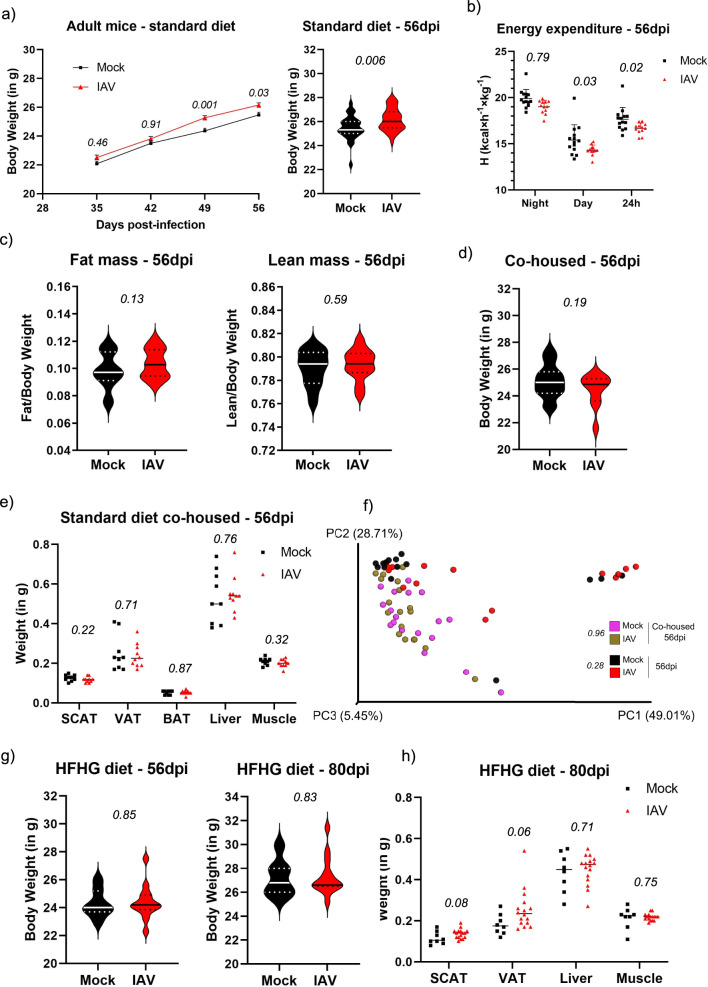
IAV related imprinting causes long term metabolic changes. **a** Body weight was monitored for each mouse weekly for 56 days after infection and values of male mice are plotted as mean ± SEM – left panel. Individual weight values of male mock-treated (*n* = 29) and IAV-infected mice (*n* = 38) at 56dpi are shown—right panel. Statistical significance between groups was determined by unpaired student’s *t*-test (left panel) and two-way ANOVA (right panel). **b** Energy expenditure in calories (H) for individual male mice at 56dpi from mock-treated (black squares) and IAV-infected groups (red triangles) are depicted for indicated periods of the day. Mean ± SD are represented. **c** Fat mass (left panel) and lean mass (right panel) of individual male mock-treated mice (*n* = 14) or male IAV-infected mice (*n* = 12) was measured by EcoMRI at 56dpi. **d** Individual weight values of male mock-treated (*n* = 15) and male IAV-infected co-housed mice (*n* = 10) at 56dpi are shown. **e** Individual organs weight from mock-treated (*n* = 9) and IAV-infected co-housed mice (*n* = 10) at 56dpi are depicted by different organ/tissue. Median is represented by a black dash for each group. Statistical significance between groups was determined by unpaired student’s *t*-tests. **f** Scaled 3D principal coordinates analysis (PCoA) plots using a weighted-UniFrac distance matrix from small intestine microbiota of co-housed mock-treated or IAV-infected mice and single housed mock-treated or IAV-infected mice at 56 post-infection. Each symbol represents one individual mouse and single-housed mice data are the same as in Fig. 1f. Percentages explain variation in PC1 (x-axis), PC2 (y-axis) and PC3 (z-axis). PERMDISP statistical tests were performed and p-values are indicated in the figure and listed in Supplementary Table 1. **g** Individual weight values of male mock-treated (*n* = 7) and male IAV-infected mice (*n* = 13) at 56dpi (left panel) or 80dpi (right panel) fed a high fat/high glucose (HFHG) diet are shown. Statistical significance between groups was determined by unpaired student’s t-tests. **h** Individual organs weight from mock-treated (*n* = 8) and IAV-infected mice (*n* = 16) fed a high fat/high glucose (HFHG) diet at 80dpi are depicted by different organ/tissue. SCAT subcutaneous adipose tissue, VAT visceral adipose tissue, BAT brown adipose tissue. Median is represented by a black dash for each group. Statistical significances were determined by two-way ANOVA. *p*-values are indicated in the graphs.

In order to address if intestinal microbiota were responsible for the weight gain, we co-housed mice in parallel to non-co-housed experiments described before, which were either treated with PBS or IAV in infancy, in equal proportion, after weaning and after viral clearance and followed weight gain until nine weeks of age. Importantly, we did not observe differences in median weight gain between PBS treated and IAV infected animals (Fig. 2d) or differences in selected organ weights (Fig. 2e), implicating that exchange of microbiota with PBS treated animals could compensate for the effects by IAV infection early in life. Co-housing resulted indeed in a significantly distinct microbiota as indicated by the shift in beta diversity away from both non-co-housed IAV-infected mice (Fig. 2f). Importantly, towards the non-co-housed mock-treated mice these differences were not significant. There was no difference in beta diversity between cohoused IAV infected and mock-treated mice, confirming the successful exchange of intestinal microbiota between infected and mock-treated mice. Accordingly, we did not find major differences in the compositional data (Fig. S5 and Supplementary Table 2). This implicates that microbiota exchange with mock animals can override the IAV-caused dysbiosis even after viral clearance.

Finally, we asked, if mice, which underwent an IAV infection in infancy were more prone to diet-induced alterations of body mass. We hence fed a high fat/high glucose (HFHG) diet ad libitum from 35d post-infection onwards to IAV infected or PBS treated mice. While the overall body mass did not differ between PBS treated and infected mice on D56 or D80 post-infection (Fig. 2g), we found an increase in visceral and subcutaneous fat in adult mice (D80 post-infection), which were infected with IAV in infancy (Fig. 2h) with about 30% median effect size in VAT. This suggests that early life infections could sensitize to adulthood food-induced gain of fat mass in specific depots. The mechanistic link between the altered microbiota after early life IAV infection and the metabolic changes will require additional investigations.

While we cannot provide a detailed mechanism at this stage, we believe that our proof of principle study demonstrates that a single acute infection early in life could cause sufficient perturbation of intestinal microbiota development to provoke long-lasting changes in commensal bacterial composition and their function in host metabolism. Beyond changes in the host metabolism, early life infections could also affect other microbiome-driven processes of the host organism.

Limitations of this study: While mice are frequently used as a model system to study pathological effects of microbiota changes, they differ in many ways from humans. Microbiota compositions is substantially different^20,21^, mice are coprophagic allowing easier exchange of intestinal microbiota between cage mates^22,23^, mice have a faster aging rate and a substantially increased metabolic rate as compared to humans. Since we cannot sample small intestinal content from mice longitudinally, we can only provide snap-shots of microbiota dynamics, without the option of following the development within each animal.

We nonetheless believe that for our proof-of-principle study the mouse model remains useful. IAV-caused microbiota changes were also described in adult human patients^24^. Association studies in humans would be required to prove, if severe childhood infections could have long-lasting impact on microbiota composition and host physiology.

## Methods

### Animal experiments, metabolic phenotyping, and organ collection

C57BL/6J mice (male/female, 7-8 weeks of age) were purchased as breeders from Charles River Laboratories (France). Animals were housed at specific pathogen free (SPF)/biosafety level 2 (BSL2) animal facility of Central Medical University (CMU) at University of Geneva under a strict 12-hour light/dark cycle and fed ad libitum with standard chow diet (RM3 (E) FG, 3 Special Diets Service, UK) unless otherwise stated. Breeding couples were time-mated and 7-day old pups, born in house to IAV naïve parents, were used for the experiments indicated in this study. At age 21d, pups were weaned and housed separately based on sex and experimental group. All animals were introduced to a new clean cage with fresh bedding and enrichment every two weeks.

For infection, 7-day-old pups were inoculated with 5 µl of PBS (1X) or IAV A/Viet Nam/1203/2004 (VN/1203, 40 pfu) HALo^10^ (low pathogenic version) through intranasal route without anesthesia. Body weights of the animals were followed daily for two weeks following infection, then weekly up to 56 days post-infection, if not every two days for animals under HFHG diet.

For physiological and metabolic phenotyping^18^, 56-day-old mice (either mock-treated or IAV infected at 7-day of age), were individually introduced into TSE Labmaster/Phenomaster (TSE Systems, Germany). Animals were adapted to the caging conditions for two days. Experimental data were gathered for 3 days following the adaptation period. Energy expenditure was calculated based on V_O2_ and V_CO2_ from individually housed mice^19^.

At 42 days post-infection, animals were introduced to clean cages in pairs. Food intake and feces production over 48 hours were measured for each cage. Fecal pellets were subsequently collected, vacuum-dried, and grounded to fine powder. Caloric content of food and feces was measured by an oxygen bomb calorimeter (Parr, 6100, USA)^25^. Average caloric uptake of two animals was calculated based on caloric content of consumed food and excreted feces. At 56 days post-infection, body composition of the animals, i.e., fat mass, lean mass, and free fluids, were analyzed through the use of EchoMRI-700 (EchoMRI, Houston, USA), without anesthesia.

For experiments including high caloric diet, animals were fed HFHG diet (HCD), (22.1 MJ/kg Gross Energy; 45 kJ% Fat, 20 kJ% Protein, 35 kJ% Carbohydrates, D12451, Ssniff, Germany) for 3 weeks starting from 35 days post-infection, at age of 6 weeks.

Upon reaching experimental endpoints, animals were euthanized using controlled CO_2_ exposure. Organs were sampled following euthanasia using sterile tools, sterilized between groups, under aseptic conditions. Tissue samples were collected from visceral adipose tissue (epididymal WAT, unilateral), subcutaneous adipose tissue (inguinal WAT, unilateral), liver (medial lobe), or muscle (quadriceps, unilateral). Samples were weighed where necessary and immediately stored at −80˚C to be used in viral titer determination, metabolic processing or extraction of DNA. Fecal pellets were freshly sampled on the day indicated before euthanasia. Infectious virus particles in indicated organs were quantified by standard plaque assay on MDCK cells^26^. Briefly, organ homogenates were precleared using 2000 × g centrifugation at 4 °C. Supernatants were serially diluted and transferred to confluent monolayers of MDCK cells. These were subsequently overlayed with 2% agar-containing medium to limit free viral diffusion and allow plaque formation. Plaques were stained with crystal violet solution and counted to determine infectious virus titers.

All animal procedures were in accordance with federal regulations of the Bundesamt für Lebensmittelsicherheit und Veterenärwesen (BLV) Switzerland (Tierschutzgesetz) and approved by cantonal authorities (License number: GE/159/17).

### Quantitative PCR for viral genomic RNA and host mRNA

Total tissue RNA was isolated using Trizol (Invitrogen). By reverse transcription (Superscript III) using either a vRNA-specific reverse primers for IAV^27^ or oligo dT primer we generated cDNA according to the manufacturer’s protocol. Quantitative PCR was performed using SYBRGreen. Primers for browning-related genes were previously published^28^. 16S and 18S specific qPCRs were described earlier^6^ (16S_F: 5′– TCCTACGGGAGGCAGCAGT -3′; 16S_R: 5′- GGACTACCAGGGTATCTAATCTT -3′; 18S_F: 5′ – GTAACCCGTTGAACCCCATT -3′; 18S_R: 5′- CCATCCAATCGGTAGTAGCG -3′; M1_F: 5′- AGATGAGTCTTCTAACCGAGGTCG -3′; M1_R: 5′- TGCAAAAACATCTTCAAGTCTCTG -3′; IP-10_F: 5′- TTCACCATGTGCCATGCC -3′; IP-10_R: 5′- GAACTGACGAGCCTGAGCTAGG -3′; IL28_F: 5′- GTTCAAGTCTCTGTCCCCAAAA -3′; IL28_R: 5′- GTGGGAACTGCACCTCATGT -3′; PRDM16-F: 5′-CAGCACGGTGAAGCCATTC-3′, PRDM16-R: 5′-GCGTGCATCCGCTTGTG-3′; UCP1_F: 5′- CACCTTCCCGCTGGACACT -3′; UCP1_R: 5′- CCCTAGGACACCTTTATACCTAATGG -3′; TBX1_F: 5′-

CIDE-A_F: 5′- TGACATTCATGGGATTGCAGAC -3′; CIDE-A_R: 5′- GGCCAGTTGTGATGACTAAGAC -3′). Samples were measured in technical triplicates. Fold changes were determined based on the 2^-ΔΔCT^ method^29^.

### Bacteria DNA Extraction, Library construction, and Bioinformatic analysis

Total DNA extraction from small intestine and fecal pellets was performed using QIAGEN Pathogen Cador Mini kit (USA) and PowerLyzer PowerSoil DNA isolation kit (MoBio, QIAGEN, USA), respectively, according to manufacturer’s instructions with slight modifications^6,30^, together process matched control tubes. DNA preps were used either for 16S rRNA DNA quantification or library preparation for analysis of bacterial composition^30^. QIIME1^31^ was used for bioinformatics analysis of the sequences generated through Illumina (USA) sequencing through a pre-defined pipeline^6,32–40^.

### Statistics

In order to determine statistical significance, we applied unpaired students’ t-test for parametric comparison of two experimental groups, or Kruskal-Wallis or 2-way Anova for comparison of longitudinal data sets, and multiple corrected t-tests for comparison of more than two parameters from the same two experimental samples, using Graph Pad Prism 7.0. T Statistical tests are indicated in each figure legend. Analysis of microbiome composition was performed using LefSe^12^. Statistical analysis of beta diversity was done with PERMANOVA and PERMADISP2^41,42^.

## Supplementary information

Supplementary Figures and Tables

## Data Availability

NGS sequencing data are deposited under NCBI Bioproject PRJNA768309. Material is available through the lead contact.
