# Supplementary material for: A single respiratory tract infection early in life reroutes healthy microbiome development and affects adult metabolism in a preclinical animal model
Source: NPJ Biofilms Microbiomes. 2022 Jul 2;8:51. doi: 10.1038/s41522-022-00315-x (PMC9250495; doi:10.1038/s41522-022-00315-x)
Supplement: Supplementary file 1 — Supplementary Figures and Tables [file 41522_2022_315_MOESM1_ESM.pdf]

Supplementary Figures

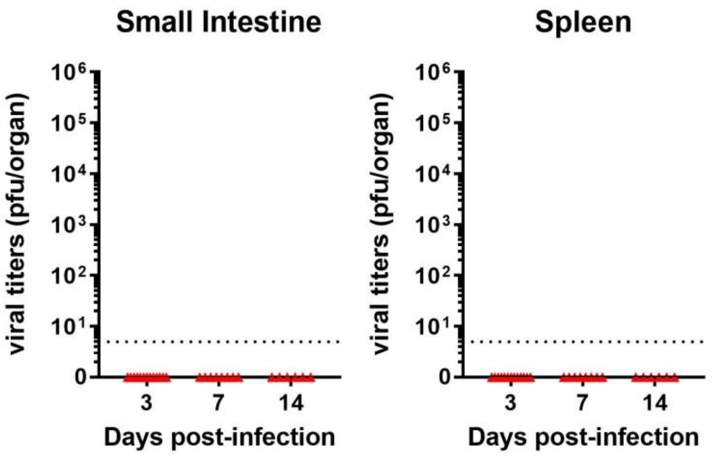

Supplementary Figure 1: IAV titers in small intestine and spleen
Seven-days-old mice were infected intranasally with PBS or 40 pfu of VN/1203 in 5 µL. (a) At 3, 7 and 14 days post-infection (dpi) viral titters in intestine (left panel) and spleen (right panel) for IAV infected mice were determined by plaque assay. Limit of detection is represented by a black dashed line. No statistical testing was performed.

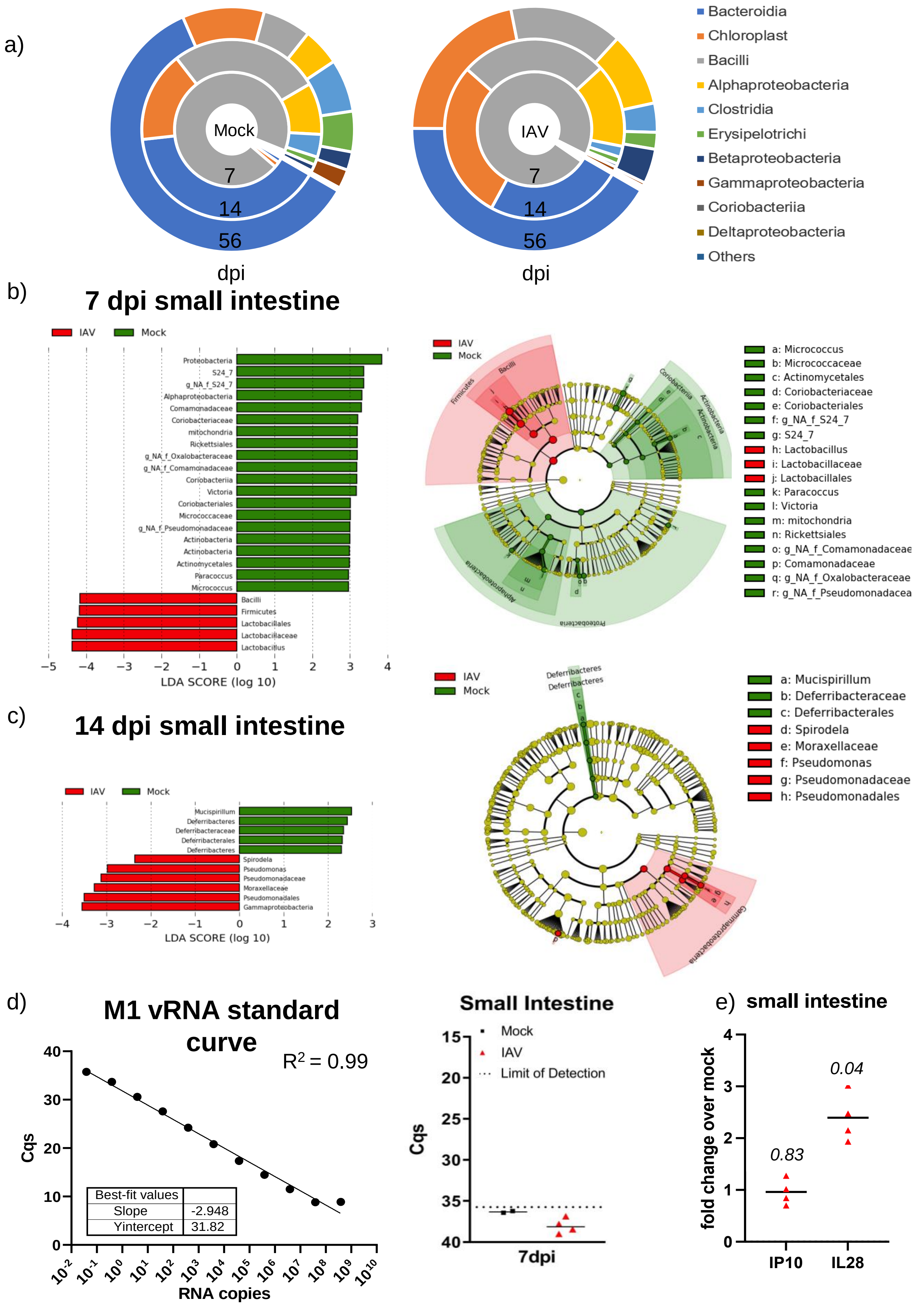

Supplementary Figure 2: microbiota composition in in small intestine

(a) Microbiota composition in the small intestine of IAV or mock treated animals on D56 post infection. Mean relative bacterial abundance as analyzed by Qiime1 is plotted for each group at class level (%). (b/c) LefSe analysis of the composition of the microbiota in small intestines of mock-treated and IAV-infected mice based on 16S rRNA gene sequencing of samples collected at day 7 post-infection (b) or day 14 post-infection (c). Linear discriminant analysis (LDA) scores for significantly overrepresented ($p<0.05$) taxonomic groups of microbiota from IAV-infected or mock-treated mice are plotted on a genus-level resolution for each group (n=12-16). Cladograms of OTUs, as annotated by Qiime1, that are differentially represented in small intestine samples taken from mock and IAV infected animals at 7 (b) or 14 (c) post-infection are shown on the right-hand side. Kruskall-Wallis statistical test was performed as described previously. Overrepresented taxonomy groups are given on legends next to the corresponding cladogram. (d) Quantitative PCR for genomic RNA of IAV in small intestine. Standard curve on the left-hand side, correlating viral RNA copies with PCR cycles (Cqs). qPCR result from small intestinal samples of mock treated mice (n=2) or IAV infected mice (n=4). Limit of detection indicated by dotted line. (e) Relative gene expression as quantified by RT-qPCR from small intestinal samples of IAV-infected (n=4) and mock-treated (n=2) animals. Statistical significance between mock and IAV groups for each gene was tested by unpaired t-test. P-values are indicated.

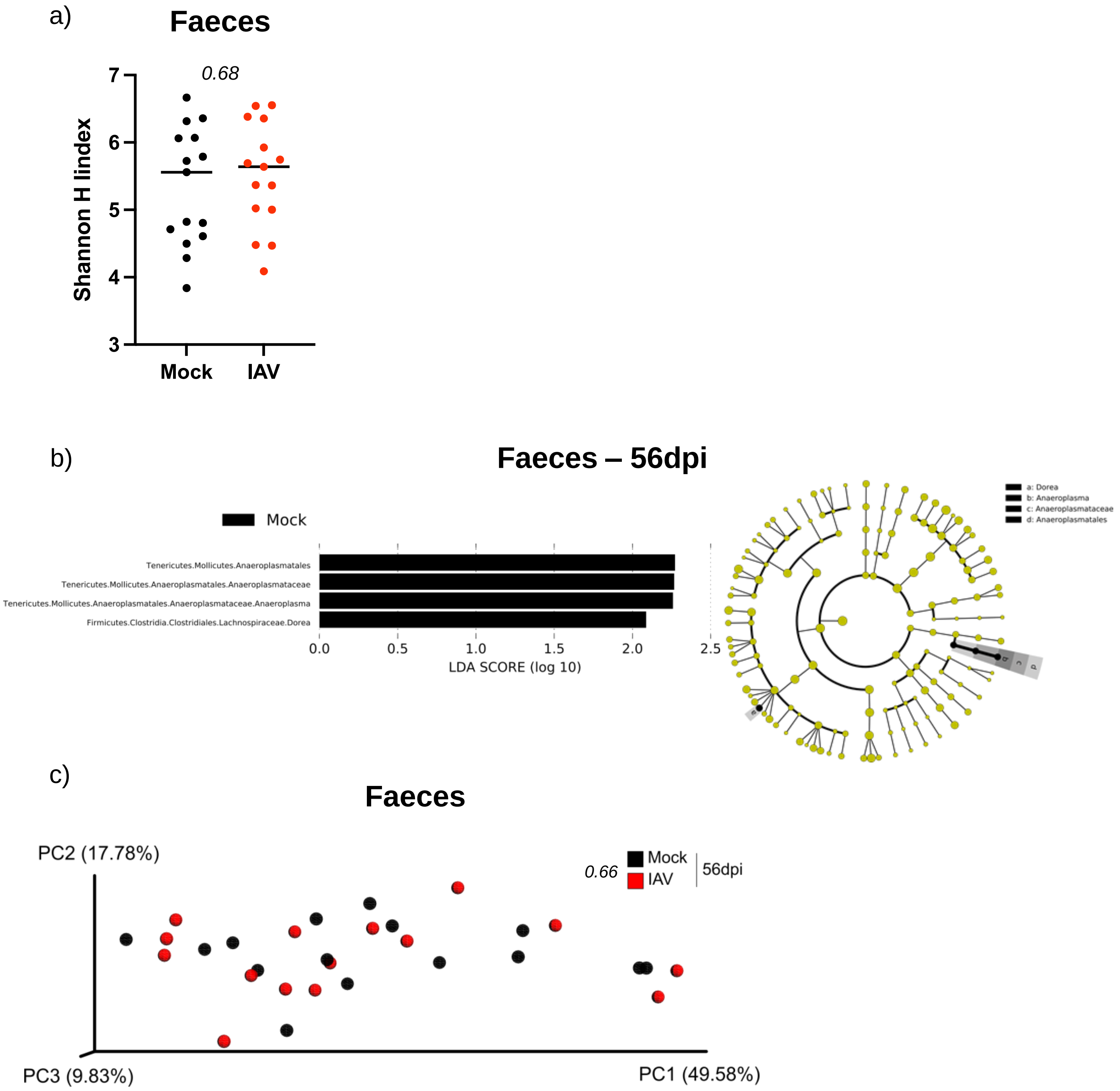

Supplementary Figure 3: microbiota composition and diversity in feces

(a) Individual Shannon H-index of feces microbiota at 56dpi of mock-treated (black squares) and IAV-infected mice (red triangles). Mean ± SD per experimental group is shown.(b) LefSe analysis of microbiota abundance in faeces of mock-treated and IAV-infected mice was determined based on 16S rRNA gene sequencing of samples collected at 56 days post-infection. LDA scores for significantly overrepresented (*p<0.05*) groups are plotted on a genus level resolution for each group (n=15). Cladograms of OTUs, as annotated by Qiime1, that are differentially represented in faeces samples taken from mock and IAV infected animals at 56 days post-infection are shown on the right-hand side. Kruskall-Wallis statistical test was performed as described previously. Overrepresented taxonomy groups are given on legends next to the corresponding cladogram.(c) Scaled 3D principal coordinates analysis (PCoA) plots using a weighted-UniFrac distance matrix from faeces microbiota of mock-treated or IAV-infected mice at 56 days post-infection. Each symbol represents one individual mouse. Percentages explain variation in PC1 (x-axis), PC2 (y-axis) and PC3 (z-axis). PERMANOVA statistical test was performed and p-value is indicated in the figure.

Supplementary Figure 4: Female mice body weight and metabolic phenotyping of IAV-imprinted adult male mice

Seven-days-old mice were infected intranasally with PBS or 40 pfu of VN/1203 in 5 µL. (a) Individual weight values of female mock-treated (n = 9) and IAV-infected mice (n=15) at 56dpi are shown. Home cage activity as recorded by TSE Labmaster/Phenomaster for different periods of the day. Each bar represents mean ± SEM of mock-treated male mice (n=14) or IAV-infected male mice (n=12) (b) Ambulatory activity. (c) Respiratory exchange ratio. (d) Fatty acid oxidation. (e) Food intake. (f) Water intake. (g) Caloric uptake of mock-treated (n = 12) and IAV-infected mice (n=12) at 42dpi was calculated based on caloric content of consumed food and excreted feces and depicted as energy consumption in kcal. Each symbol represents the average of two mice. Statistical significances were determined using unpaired t-test (panel a and g), 2-way Anova (panel b-f), or multiple t-tests (panel h). P-values are indicated in each panel.

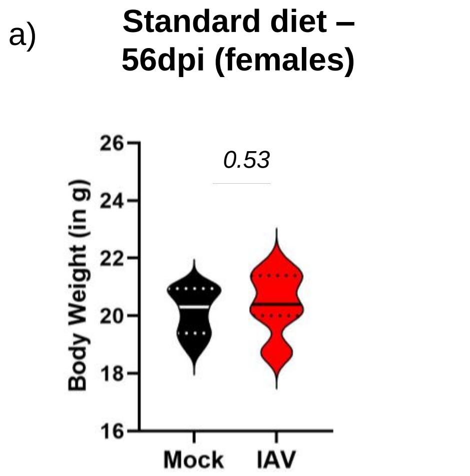

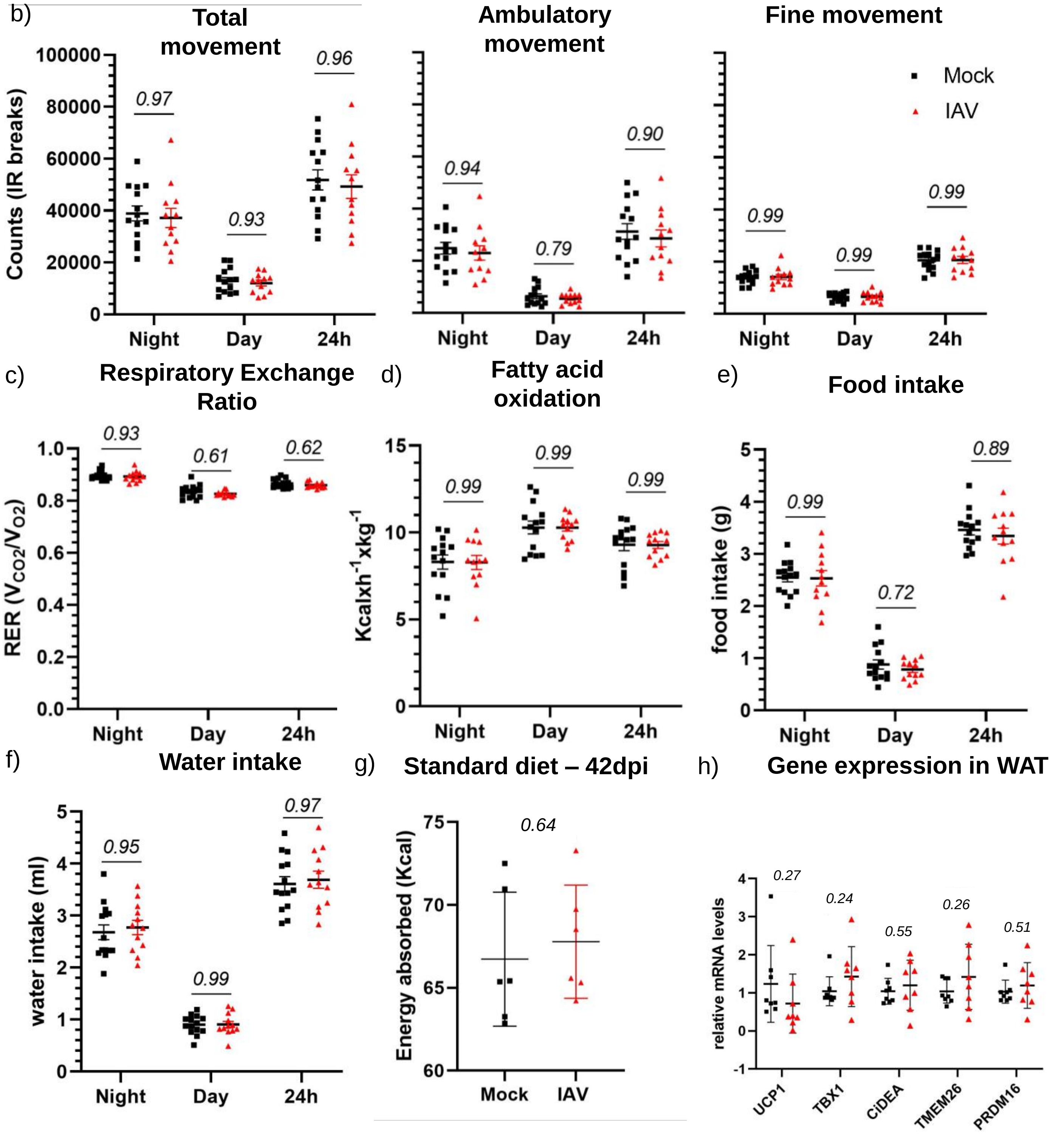

coMock vs coIAV

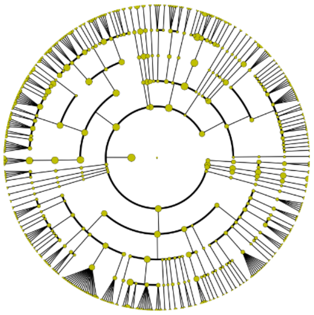

Mock vs coMock

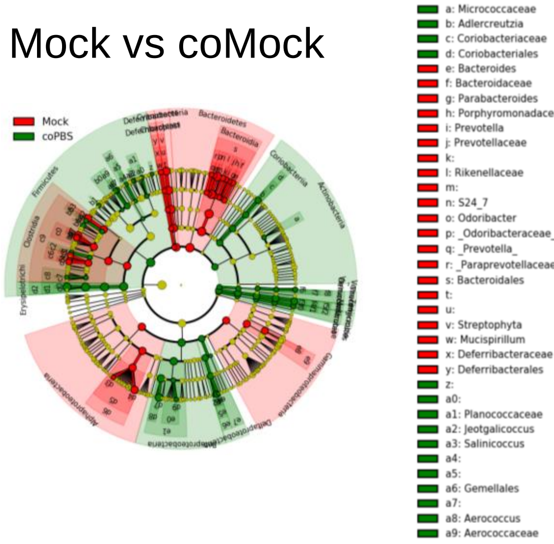

Mock vs coIAV

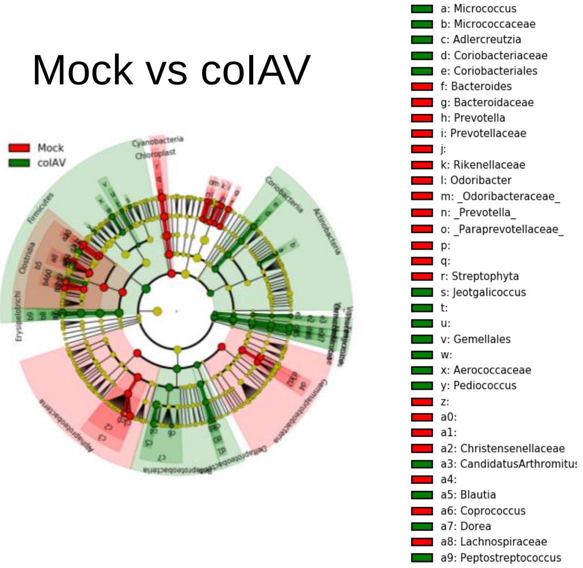

IAV vs coMock

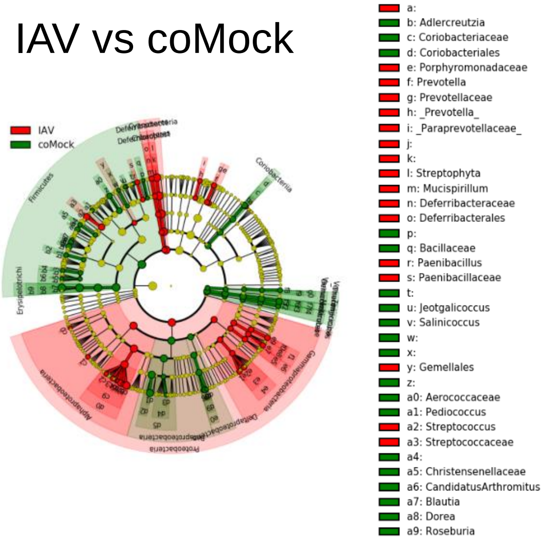

IAV vs coIAV

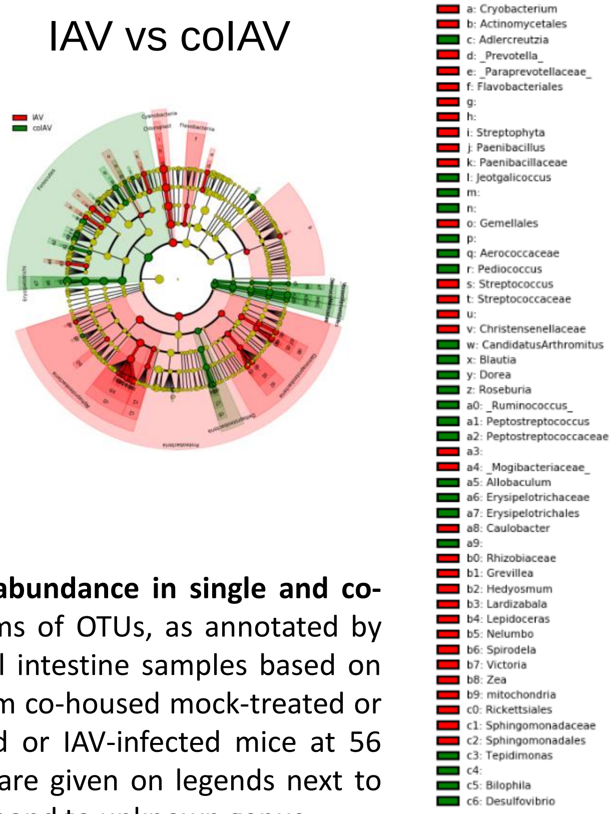

Supplementary Figure 5: Differential microbiota abundance in single and co-housed mice treated with IAV or mock Cladograms of OTUs, as annotated by Qiime1, that are differentially represented in small intestine samples based on LDA scores. Small intestine samples were taken from co-housed mock-treated or IAV-infected mice and single housed mock-treated or IAV-infected mice at 56 post-infection. Overrepresented taxonomy groups are given on legends next to the corresponding cladogram. Blank legends correspond to unknown genus.

Supplementary Tables

Supplementary Table 1: Statistical test of PCoA. PERMADISP analysis for direct comparison of indicated data sets from Figure 1f resulted in displayed p-values.

PERMDISP						
p-value						
	IAV_14dpi	IAV_56dpi	IAV_7dpi	Mock_14dpi	Mock_56dpi	Mock_7dpi
IAV_14dpi		0.0390	0.0010	**0.9640**	0.8740	0.0010
IAV_56dpi	0.0315		0.0010	0.0320	**0.2310**	0.0010
IAV_7dpi	<0.0001	<0.0001		0.0010	0.0050	**0.0800**
Mock_14dpi	0.9619	0.0289	<0.0001		0.8450	0.0010
Mock_56dpi	0.8630	0.2331	0.0044	0.8521		0.0070
Mock_7dpi	<0.0001	<0.0001	0.0988	<0.0001	0.0057	

method name	**PERMANOVA**
test statistic name	pseudo-F
sample size	30
**number of groups**	**2**
test statistic	1.657099606
**p-value**	**0.178**
number of permutations	999

Supplementary Table 2: Statistical test of PCoA. PERMADISP analysis for direct comparison of indicated data sets from Figure 2f resulted in displayed p-values.

PERMDISP				
p-value				
	coIAV	coPBS	IAV_56dpi	Mock_56dpi
coIAV		**0.961**	0.002	0.396
coPBS	0.964		0.002	0.389
IAV_56dpi	0.003	0.004		**0.283**
Mock_56dpi	0.365	0.385	0.274	
